# Age-dependent contribution of intrinsic mechanisms to sinoatrial node function in humans

**DOI:** 10.1038/s41598-023-45101-7

**Published:** 2023-11-01

**Authors:** Ido Weiser-Bitoun, Hitoshi Mori, Taisuke Nabeshima, Naomichi Tanaka, Daisuke Kudo, Wataru Sasaki, Masataka Narita, Kazuhisa Matsumoto, Yoshifumi Ikeda, Takahide Arai, Shintaro Nakano, Naokata Sumitomo, Taka-aki Senbonmatsu, Kazuo Matsumoto, Ritsushi Kato, Christopher H. Morrell, Kenta Tsutsui, Yael Yaniv

**Affiliations:** 1 Faculty of Biomedical Engineering, Technion–Israel Institute of Technology, Haifa, Israel; 2 Rappaport Faculty of Medicine, Technion–Israel Institute of Technology, Haifa, Israel; 3 Saitama Medical University International Medical Center, Saitama, Japan; 4 Laboratory of Cardiovascular Science, Intramural Research Program, National Institute on Aging, National Institutes of Health, Baltimore, MD USA; 5 Department of Cardiovascular Medicine, Saitama Medical University International Medical Center, Saitama, Japan; 6 Laboratory of Bioenergetic and Bioelectric Systems, The Faculty of Biomedical Engineering Technion—IIT, Haifa, Israel

**Keywords:** Cardiology, Health care, Medical research

## Abstract

Average beat interval (BI) and beat interval variability (BIV) are primarily determined by mutual entrainment between the autonomic-nervous system (ANS) and intrinsic mechanisms that govern sinoatrial node (SAN) cell function. While basal heart rate is not affected by age in humans, age-dependent reductions in intrinsic heart rate have been documented even in so-called healthy individuals. The relative contributions of the ANS and intrinsic mechanisms to age-dependent deterioration of SAN function in humans are not clear. We recorded ECG on patients (n = 16 < 21 years and n = 23 41–78 years) in the basal state and after ANS blockade (propranolol and atropine) in the presence of propofol and dexmedetomidine anesthesia. Average BI and BIV were analyzed. A set of BIV features were tested to designated the “signatures” of the ANS and intrinsic mechanisms and also the anesthesia “signature”. In young patients, the intrinsic mechanisms and ANS mainly contributed to long- and short-term BIV, respectively. In adults, both ANS and intrinsic mechanisms contributed to short-term BIV, while the latter also contributed to long-term BIV. Furthermore, anesthesia affected ANS function in young patients and both mechanisms in adult. The work also showed that intrinsic mechanism features can be calculated from BIs, without intervention.

## Introduction

While basal heart rate is not affected by age in humans^1^, age-dependent reductions in intrinsic heart rate have been documented even in so-called healthy individuals^2^. In severe conditions, sick sinus syndrome, also known as sinoatrial node (SAN) dysfunction (SND), is observed. This disorder is commonly observed in patients older than 75 years^3^ or young patients with genetic variants^4–6^. SND diagnosis is challenging and is mainly performed indirectly, using electrocardiography (ECG), Holter monitoring and exercise test.

The autonomic nervous system (ANS) and the internal “coupled-clock” (i.e. intrinsic) system in SAN tissue are the two main mechanisms that control beat interval (BI) dynamics and drive the reduction in intrinsic pacemaker function in apparently healthy individuals and in patients with SND^7,8^. The ANS stimulates receptors on the SAN membrane, which subsequently activate internal pacemaker signaling cascades by affecting cAMP/PKA signaling. cAMP/PKA signaling activates channels on the cell membrane and internal clocks that control local Ca^2+^ release from internal storage, which, in turn, triggers Ca^2+^-dependent channels and exchangers to trigger an action potential^9^.The same signaling cascades can be activated by the internal-cellular “coupled-clock” system which is primarily activated by Ca^2+^ and other associated ions^10^ without ANS stimulation^11^. Taken together, the ANS and intrinsic mechanisms mutually entrain SAN function^12,13^. Because reductions in intrinsic heart rate can occur at different ages (etiology-dependent), it is important to understand the age-dependent relative contribution of the ANS and intrinsic SAN mechanisms to SAN function.

According to the European Society of Cardiology (ESC) and American Heart Association (AHA) guidelines^14,15^, intrinsic heart rate, without the ANS contribution, can be measured by temporarily blocking the ANS by administering a combination of atropine and propranolol, which respectively block cholinergic and beta-adrenergic receptor stimulation on the SAN membrane. However, there are two main obstacles for this method. The first is related to the anesthesia that in some cases, is administered during such procedures^2,16^, which may affect the ANS function and/or intrinsic SAN mechanisms. In addition, if such an effect exists, it is unknown if it is age-related. Furthermore, the invasive use of drugs is not a routine practice.

By examining the average BI without the use of ANS blockade, it is impossible to determine whether SND is a result of SAN tissue deterioration and/or changes in the ANS stimulation on the SAN tissue. Note, that the average BI is calculated by averaging the length of the beat intervals in a certain time interval. However, in the human heart, the BI changes on a beat-to-beat basis^17^. We have shown that the relative contribution of the ANS and intrinsic SAN mechanisms to the SAN function can be quantified by analyzing long- and short-term beat interval variability (BIV). This method was tested on dogs^11^, rabbits and mice^18^, but only a proof of concept was performed on humans^11^. Moreover, the age-dependent contribution of ANS and intrinsic SAN mechanisms to SAN function and to changes in BIV are not known. Understanding the mechanisms underlying SAN function and dysfunction will enhance diagnosis and treatment of cardiac pathologies. Clear and fast diagnosis of SND in the young with possible genetic etiology and the aged population will facilitate therapy optimization and prevent complications that can accompany genetic and age-dependent SAN dysfunction, such as heart failure, aortic dissection and sudden cardiac death.

We hypothesized that there is an age-dependent shift in the relative contribution of the ANS and intrinsic SAN mechanisms to SAN function. We further hypothesized that propofol and dexmedetomidine anesthesia affect the ANS and intrinsic SAN mechanisms and this effect is age-dependent. Finally, we hypothesized that specific features, which represent the “signatures” of the ANS and intrinsic SAN mechanisms in BI signals, can be obtained from BIV analysis derived from standard electrocardiogram (ECG) recordings.

To assess the age-dependent shift in the relative contribution of the ANS and the internal “coupled-clock” mechanisms to SAN function, we analyzed ECG signals from 16 young and 23 adult patients collected in the basal state and in response to pharmacological denervation, also known as “neural double blockade”. BIV measurements were analyzed and compared to determine the relative contributions of intrinsic SAN and ANS and to define a set of features, designated the “signatures” of these systems.

## Results

### BIV in young patients

**Figure 1:**
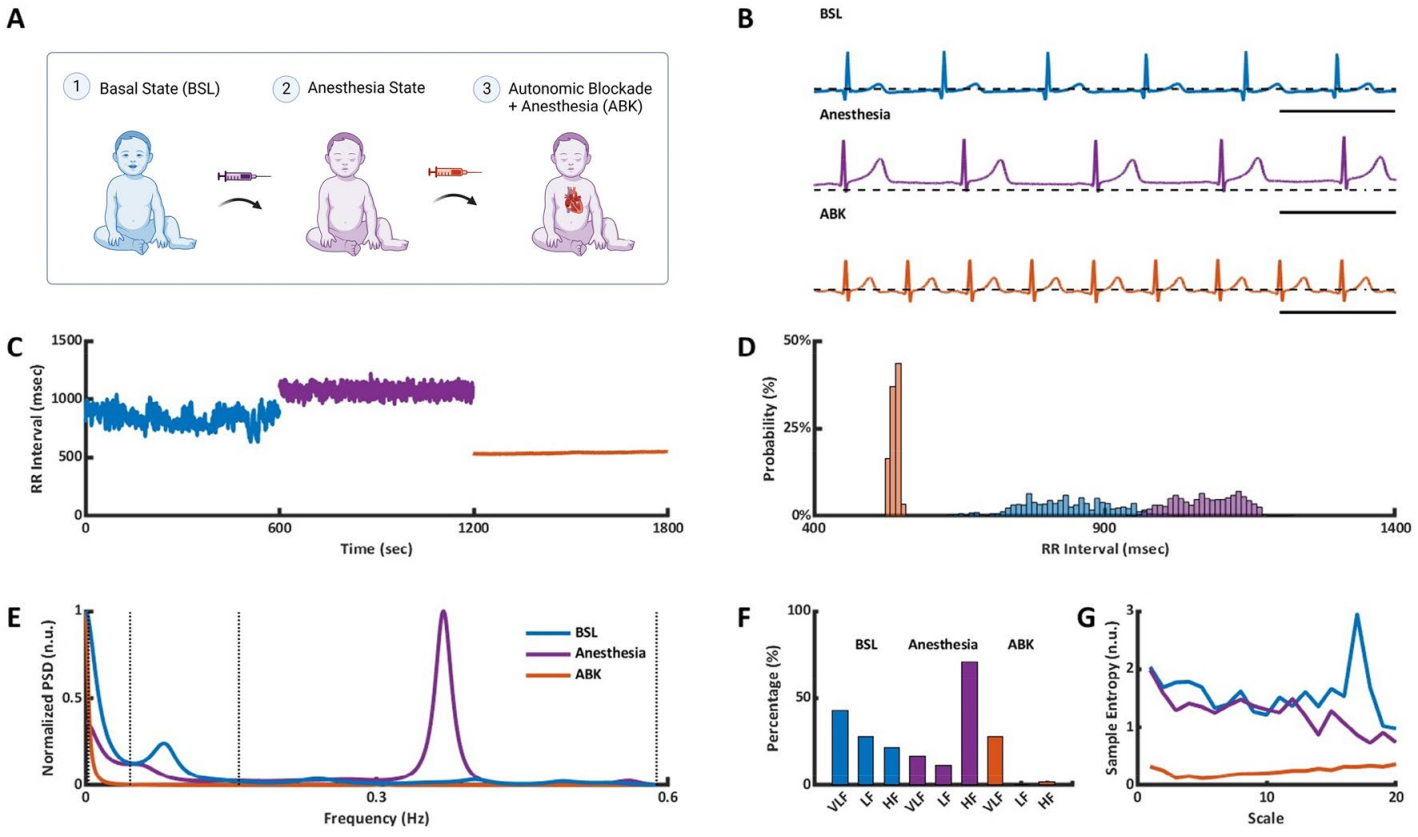
Representative example of ECG data and BIV analysis of a young patient. (**A**) Data acquisition process, which included ECG Holter recording in basal state (BSL), in anesthesia state and following autonomic blockade under anesthesia (ABK). Representative example of (**B**) ECG recordings, (**C**) intervalogram, (**D**) beat interval histogram, (**E**) normalized power spectral density (PSD), (**F**) frequency bands and (**G**) multiscale entropy (MSE) in all three states.

ECG recordings from 16 patients aged 5–19 years (with an average of 16 ± 11 years denoted as young) were included in the analysis. Six contained 24-h Holter recordings (denoted as *BSL*) and at least 10-min segments under propofol and dexmedetomidine anesthesia (denoted as *Anesthesia*), 2 contained an anesthesia segment followed by ANS blockade (denoted as *ABK*) in the presence of anesthesia, and 8 contained all three segments (see “Methods” section) (Fig. 1A). A wide range of BIV metrics were calculated from the ECG recordings using the PhysioZoo platform^19^. Various BIV metrics were used to quantify physiological complexity using time-domain, frequency-domain, and nonlinear methods^20^.

The average BI was extended from 680 ± 35 ms in the basal state to 987 ± 43 ms in the presence of anesthesia (Table S1) and was shortened to 572 ± 18 ms after ANS blockade (Table S1), shorter than the BSL. Thus, when comparing the Anesthesia and ABK states, it can be concluded that ANS activity prolongs the average BI duration, while intrinsic SAN activity, without ANS influence, shortens it.

Figure 1 presents the changes in select BIV metrics on shift from the basal state to Anesthesia, and ABK in a representative patient (Fig. 1B). Figure 1C,D show that the BI scattering around the mean increased under anesthesia and was lower following ANS blockade compared to the Anesthesia and BSL states. Time-domain BIV analysis found reductions in all metrics following ANS blockade as compared to the other two states (Table S1). The time-domain BIV indices are calculated on a beat-to beat basis, and thus, represent the short-term BIV. Therefore, the reduction in these metrics following ANS blockade suggests that the ANS contributes mostly to the short-term variability. Moreover, in the presence of anesthesia, the time-domain BIV analysis identified increases in RMSSD and pNN50, i.e. anesthesia increases short-term BIV.

Figure 1E,F show a representative example of power spectrum density (PSD) and the relative PSD in the very low frequency (VLF), low frequency (LF) and high frequency (HF) bands, in BSL state and in the presence of anesthesia before and after ANS blockade. On average (Table S1), anesthesia increased the relative PSD of the HF band and decreased that of the VLF band. Following ANS blockade, the relative PSD of the HF band returned to its basal level, while the relative PSD of the VLF band increased. The increase in HF under anesthesia led to a reduction in the relative PSD in the LF band and contributed to its reduction in the VLF band. This suggests that the administered anesthesia mainly contributed to the HF band. HF mainly represents the short-term BIV, which strengthens the conclusion reached from the time-domain analysis with regards to the contribution of propofol and dexmedetomidine anesthesia to short-term BIV.

When compared to the BSL state, the absolute PSD in the ABK state was reduced by two orders of magnitude over the entire frequency spectrum, suggesting that the ANS adds substantial broadband power. Comparison of the normalized PSD in each band before versus after anesthesia, found an increase in the HF and a decrease in the VLF bands (Table S1). Comparison of the normalized PSD in each band before versus after ANS blockade in the presence of anesthesia, uncovered a reduction in PSD in both the LF and HF bands, while an almost five-fold increase was observed in the VLF band. Comparison of the BSL versus ABK states with anesthesia showed a reduction in the normalized LF band, with an increase in the normalized PSD in the VLF band. When considering our finding that anesthesia contributes to the HF, these observations show that the primary spectral contribution of the intrinsic SAN is in the VLF band. The conclusion that the ANS system is the main contributor to the HF and LF bands and that anesthesia mainly effects the HF band, implies that propofol and dexmedetomidine mainly affect the ANS system.

To further support our conclusion regarding the contribution of the ANS and the effect of anesthesia, multiscale entropy (MSE) was used to quantify the degree of complexity in the BI signals^21^. Using this method, the irregularity of the signal is estimated on sequences of length m compared to connective sequence (i.e. length m + 1). Sample entropy is calculated by averaging the data points of multiple coarse-grained time series within non-overlapping windows of increasing length, or “scale”. The sample entropy graph (Fig. 1G) is plotted as a function of scale, where lower scales represent short-range and higher scales represent the long-range temporal phenomena. Table S1 shows that in the basal state, minimal loss of complexity was maintained in the highest compared to lowest scales, as expected based on previous works^22,23^. In contrast, in response to propofol and dexmedetomidine, there was an opposite trend in which highest scales (above 10) had lower entropy than the lowest scales (below 10), with a reduction in both low and high scales relative to BSL. The decreasing trend of the entropy (i.e. lower scales higher than high scales) was consistent with the results in the time and frequency domains and the finding that anesthesia mainly affects the HF band. On ABK in the presence of anesthesia, both low and high scales were reduced relative to BSL and low scales were reduced relative to anesthesia. Because the anesthesia per se decreased the high scales, the additional reduction in low scales was likely driven by the ANS inhibition.

### BIV in adult patients

**Figure 2:**
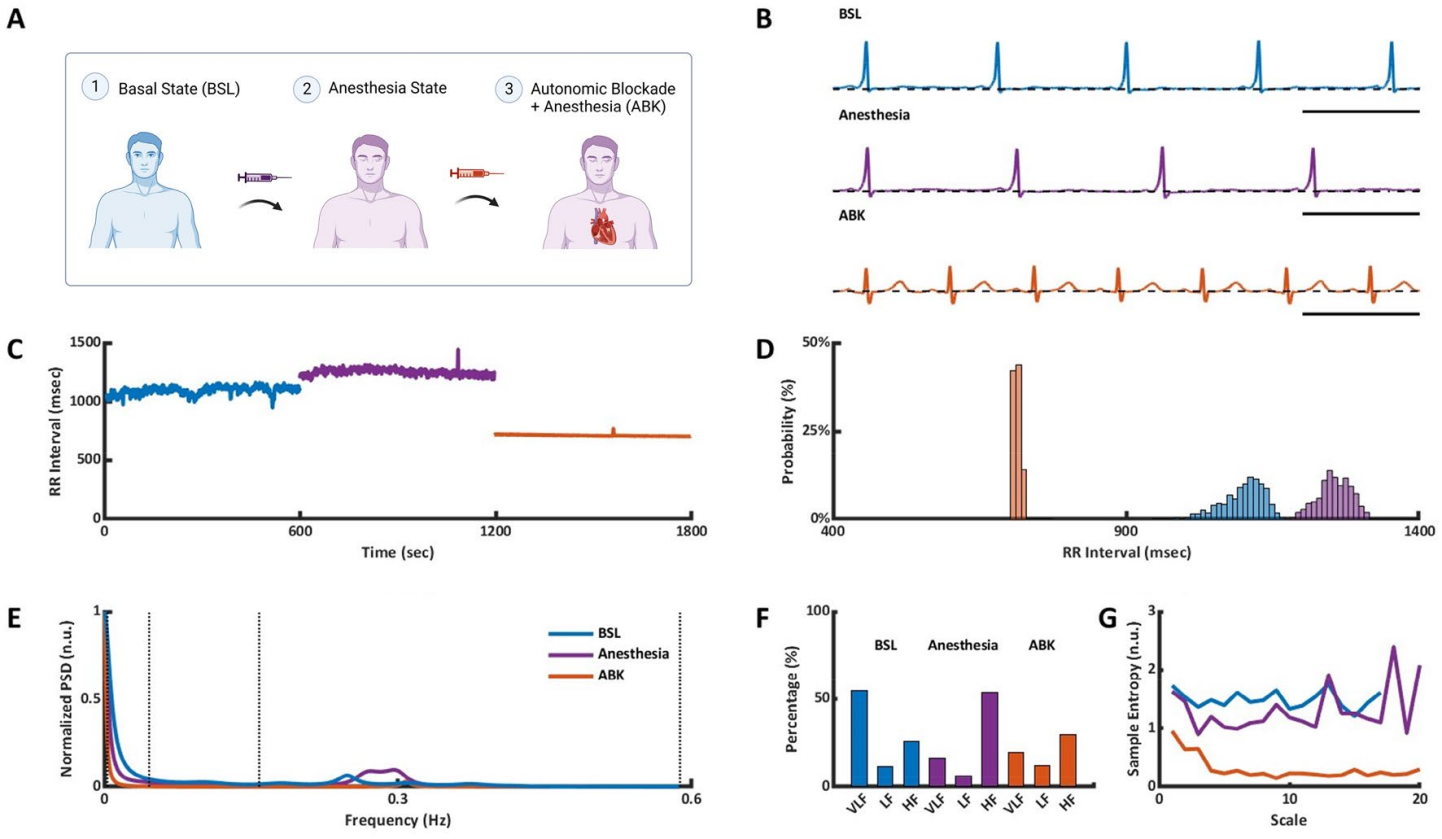
Representative example of ECG data and BIV analysis of an adult patient. (**A**) Data acquisition process, which included ECG Holter recording in basal state (BSL), in anesthesia state and following autonomic blockade under anesthesia (ABK). Representative example of (**B**) ECG recordings, (**C**) intervalogram, (**D**) beat interval histogram, (**E**) normalized power spectral density (PSD), (**F**) frequency bands and (**G**) multiscale entropy (MSE) in all three states.

ECG recordings from 23 patients aged 41–78 years with an average of 59 ± 12 years were included in the analysis. Twelve of the recordings contained at least 10-min segments in the BSL and after ANS blockade under anesthesia, 9 contained at least 10-min segments before and after anesthesia and two contained all three phases (BSL, Anesthesia and ABK), (Fig. 2A). Similar to the young, ECG analysis and BIV metrics calculation were performed.

Anesthesia prolonged the average BI from 874 ± 25 ms in the basal state to 1115 ± 39 ms while ANS blockade shortened it to 732 ± 27 ms (Table S1), shorter than the BSL. As in the young patients, when comparing the Anesthesia to ABK phases, ANS activity prolonged the average BI duration, while intrinsic SAN activity without ANS influence, shortened it.

Figure 2 presents the changes in select BIV metrics between the BSL, Anesthesia, and ABK phases in a representative patient (Fig. 2B). Figure 2C,D show that the BI scattering around the mean increased under anesthesia and was reduced following ANS blockade compared to the Anesthesia and BSL states. Time-domain BIV analysis revealed reductions in some metrics following ANS blockade compared to the two other states (Table S2). These findings suggest that the reduction in these BIV metrics following ANS blockade in all overall ages, imply the ANS contributes mostly to the short-term variability. In contrast to the young, no changes in most time-domain BIV indices were observed between BSL and Anesthesia in the adult.

Figure 2E,F show a representative example of PSD and the relative PSD in each band in the basal state and under anesthesia before and after ANS blockade. On average (Table S1), propofol and dexmedetomidine increased the relative PSD in the HF band, which led to a reduction in the relative PSD in the LF and VLF bands; namely, anesthesia contributes to both short- and long-range BIV. No change in the total power was found before versus after anesthesia, suggesting that anesthesia contributes to the HF band at all ages. However, the effect was less dominant than in the young, and with no supportive evidence in the time-domain analysis.

When comparing the BSL vs. ABK states, absolute PSD was reduced by two orders of magnitude over the entire frequency spectrum, as was found in the young. This suggests that the ANS adds substantial broadband power at all ages. Comparison of the normalized PSD in each band before and after ANS blockade in the presence of anesthesia, found a reduction in the LF band with no change in the HF and VLF bands (Table S1).

MSE was used to further inspect the data regarding the ANS and anesthesia contribution. Figure 2G and Table S1 show that, as seen in the young, under basal state, minimal loss of complexity was maintained in the highest compared to the lowest scales, as expected based on previous works^22,23^. Under anesthesia, there was a decrease in both low scales (below 10, except the first scale) and high scales (above 10). The reduction in VLF in the presence of anesthesia together with the increase in the HF band can explain these results.

### Correlation between average BI and age

**Figure 3:**
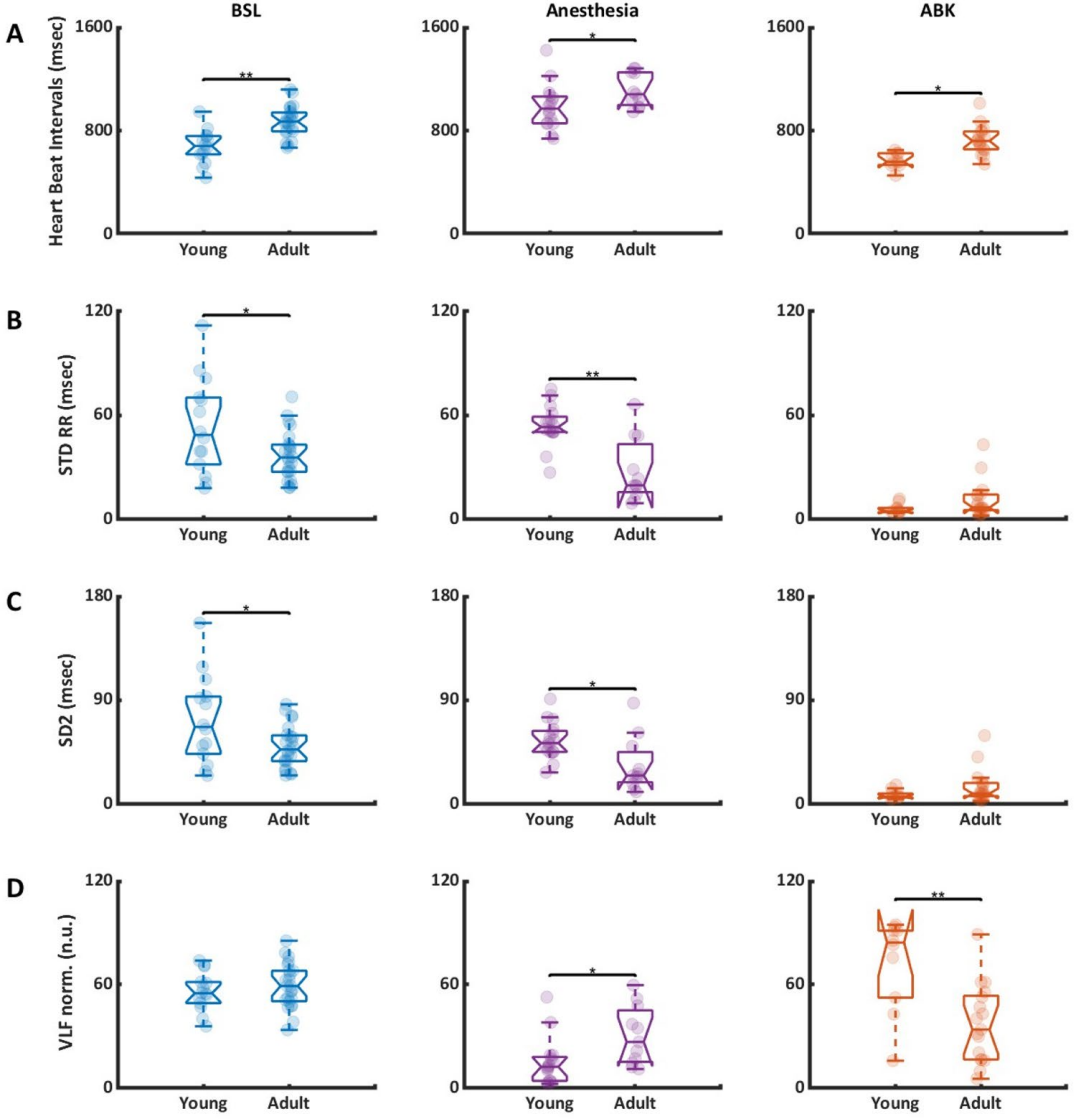
The relation between selected beat interval variability in each state in young and adult patients. (A) The average level of (**A**) beat interval (BI), (**B**) standard deviation (STD) of BIs, (**C**) SD2, and (**D**) normalized very-low-frequency (VLF) band, between each state (BSL, Anesthesia, ABK) in young and adult patients. Significant comparisons are based on FDR adjusted p-values from post-hoc pairwise comparisons from the mixed ANOVA models, *p–v < 0.05, **p–v < 0.001.

Figure 3 presents a comparison of select BIV metrics in young versus adult patients during BSL, Anesthesia, and ABK phases. Figure 3A shows that the average BI was shorter in young vs. adult patients under BI conditions, and under anesthesia before and after ABK. Figure 3B shows that the standard deviation of the BI was higher in both BSL and Anesthesia phases in young patients, while no difference between the two age groups was noted during ABK. Figure 3C shows that SD1 was similar between young and adult patients under BSL and ABK conditions, but was reduced in adult compared to young in the presence of anesthesia. Figure 3D shows that the normalized VLF band was similar for young and adult patients under basal conditions, but was higher in adults compared to young in the presence of anesthesia and decreased in adult compared to young during ABK. Because the normalized VLF band was determined by the intrinsic SAN system, a reduction in its magnitude in adult compared to young patients implies that specific human BIV parameters that are controlled by the intrinsic SAN mechanisms are age-dependent. Additional correlations appear in Table S2.

### Correlation between age and ABK BI

**Figure 4:**
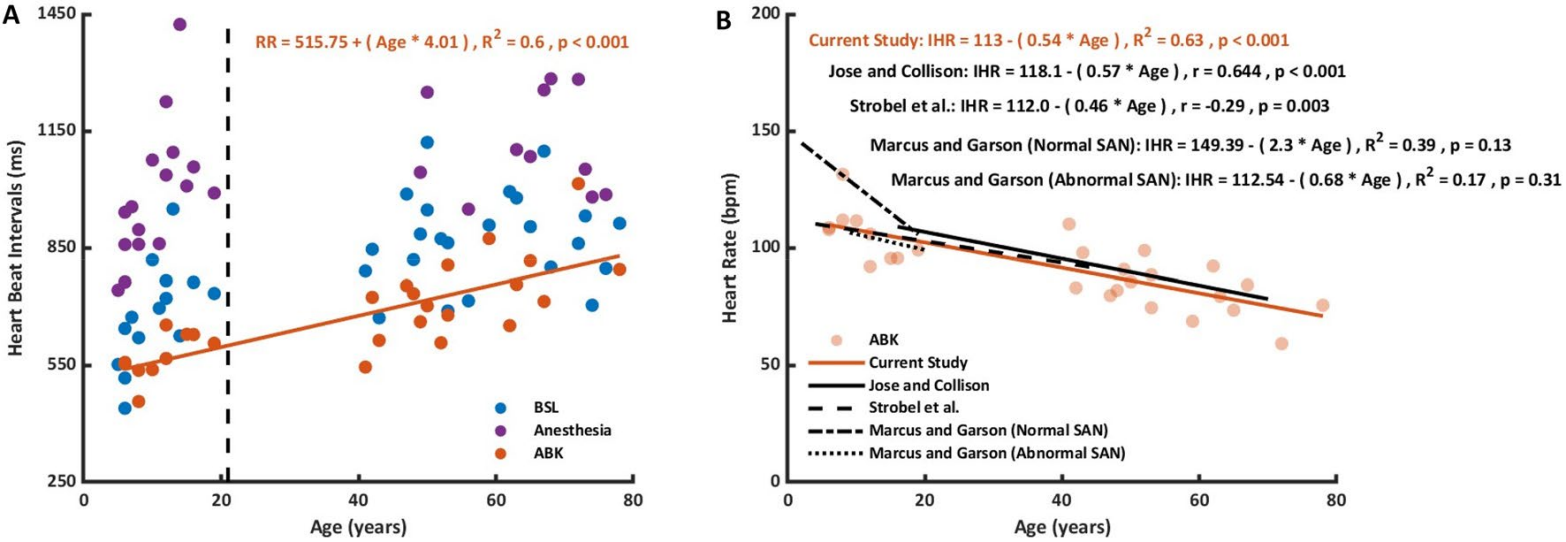
Linear association between beat interval and age. (**A**) Average beat interval (BI) as a function of age, of all recordings in basal state (BSL; blue), anesthesia (purple) and autonomic blockade under anesthesia (ABK; orange). Dashed vertical line represents 21 years of age (cut off age of the two age groups). The linear regression line represents the linear regression between BI and age. (**B**) Average heart rate as a function of age, of all ABK recordings under anesthesia (light orange). The linear regression lines were highly similar to those obtained in previous publications.

Figure 4A shows the average BI of each recording for young and adults in BSL, Anesthesia, and ABK phases. For BSL, there was no correlation between the average basal BI and age. Thus, in BSL, the average BI is not age-dependent for both young and adult, as was shown before^24^. A similar conclusion was reached for the Anesthesia condition. However, a linear age dependency was noted for the ABK conditions together with anesthesia, which corresponds to the equation: RR = 515.75 + (age * 4.01), similar to other published correlation (Fig. 4B).

### Modeling the effect of anesthesia on heart beat dynamics

**Figure 5:**
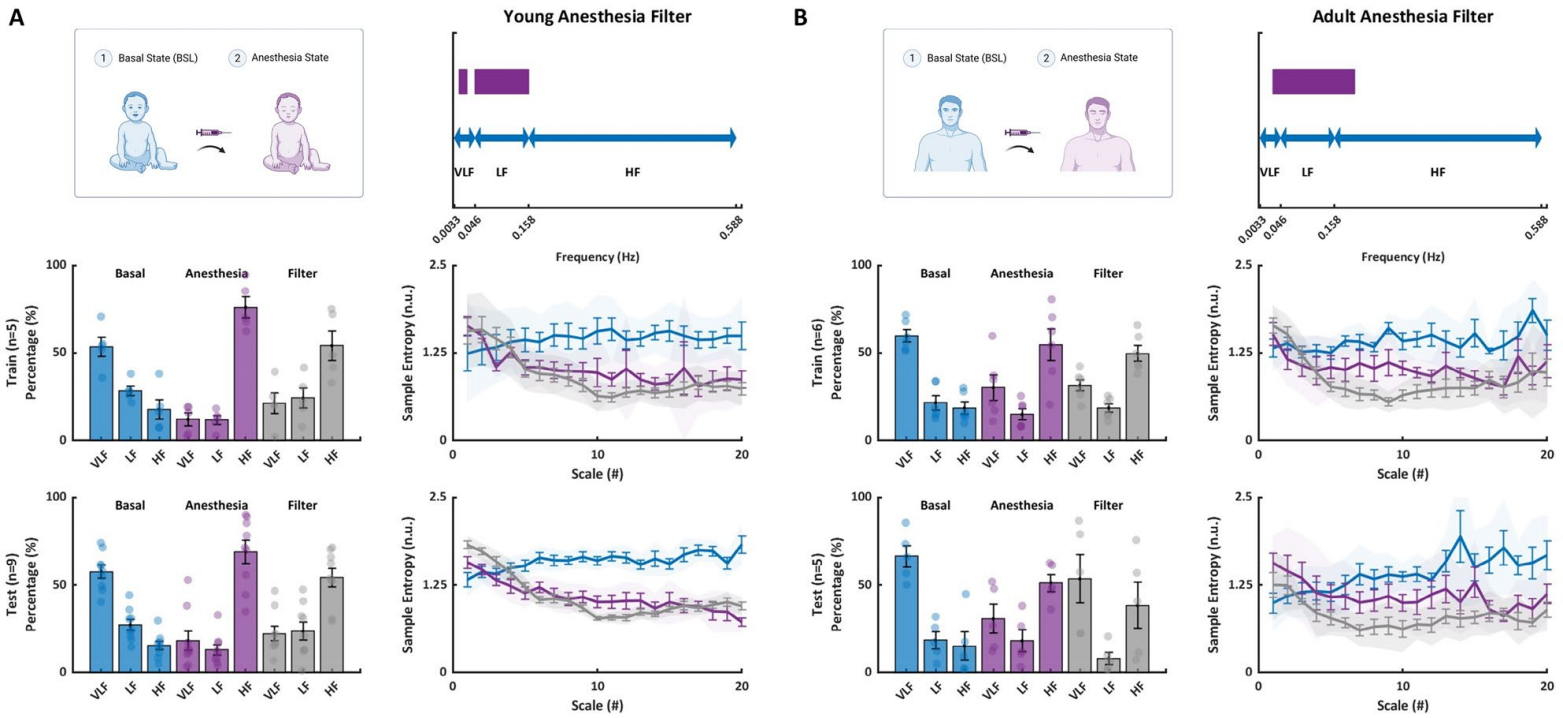
Effect of anesthesia on ANS and SAN signatures in human basal electrocardiogram recordings. Presented is the MSE at basal, anesthesia, and after filter for (**A**) young and (**B**) adult patients (bolded line represents the mean, error bars represent SE, shaded band represents 95% confidence interval). For young patients, the filter included band-pass filtering of a segment of the VLF band and all LF bands. The data were divided into train (n = 5) and test (n = 9) sets. The similarity between the anesthesia state and the filter is expressed by the high mean p-value across all scales, which was found to be 0.46 and 0.31 for train and test groups, respectively. For adult patients, the filter included a segment of the VLF band, all LF bands, and a segment of the HF band. The data were divided into train (n = 6) and test (n = 5) sets. The similarity between the anesthesia state and the filter is expressed by the high mean p-value across all scales, which was found to be 0.34 and 0.23 for train and test groups, respectively. (*ANS* autonomic nervous system, *SAN* sinoatrial node, *ECG* electrocardiogram, *MSE* multiscale entropy, *SE* standard error).

The presented findings established that in the young, anesthesia mainly contributes the short-range BIV (HF range). Herein, we present a method to simulate the anesthesia effect by filtering the PSD in the LF and VLF bands, which artificially increases the relative power in the HF (see “Methods” for details). The filter was trained on data from 5 patients and tested on filter on data from 9 patients. MSE analysis of the effect of this method showed a good fit between the anesthesia and the filtered data among MSE scales (Fig. 5A; average MSE p-values were 0.5 and 0.22 across all scales for train and test groups, respectively).

The presented findings established that the anesthesia contributes to both short- and long-range BIV in the adult. Thus, we filtered the LF band, as well as the HF band that was most affected by anesthesia (see “Methods” for details). The filter was trained on data from 6 patients and tested on data from 5 patients. MSE analysis of the effect of this method found a good fit between the anesthesia and the filtered data among MSE scales (Fig. 5B; average MSE p-values were 0.5 and 0.45 across all scales for train and test groups, respectively). Taken together, the effect of anesthesia on BIV can be artificially modeled for young and adult.

### The intrinsic SAN mechanism signature

**Figure 6:**
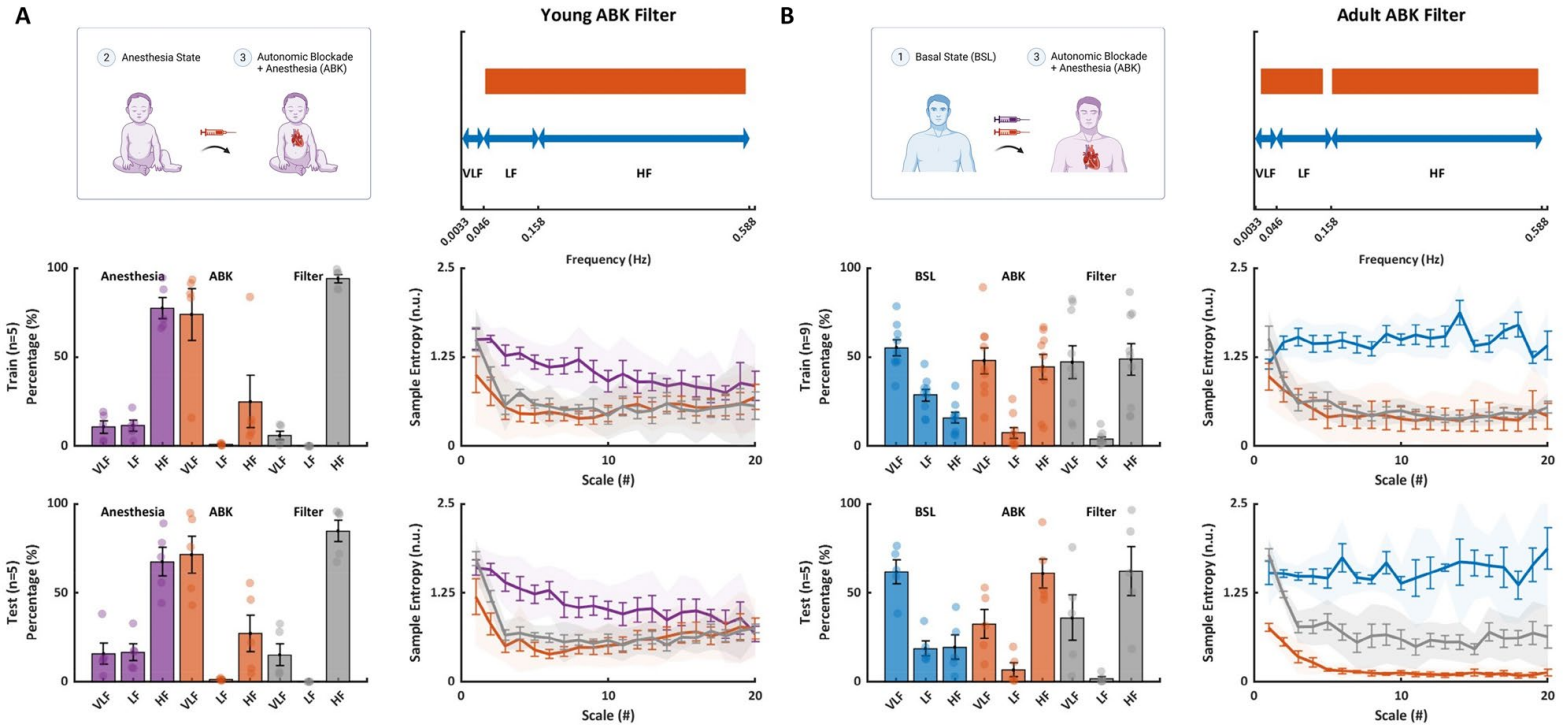
The ANS and SAN signatures in human basal ECG. (**A**) The MSE at anesthesia, autonomic blockade (ABK), and after applying a filter on the data from young patients. The filter included band-pass filtering of most of the LF band and almost all HF bands, as shown on the spectrum. The data were divided into train (n = 5) and test (n = 5) sets. The similarity between ABK state and the filter is expressed by the high mean p-value across all scales, which was found to be 0.63 and 0.55 for the train and test sets, respectively. (**B**) The MSE at basal, ABK under propofol and dexmedetomidine anesthesia, and after applying filter on the data from adult patients. The filter included most of the VLF and LF bands and almost all HF bands. The data were divided into train (n = 9) and test (n = 5) sets. The similarity between the anesthesia state and the filter is expressed by the high mean p-value across all scales, which was found to be 0.69 and 0.55 for the train and test sets, respectively. (MSE bolded line represents the mean, error bars represent SE, shaded band represents 95% confidence interval). (*ANS* autonomic nervous system, *SAN* sinoatrial node, *ECG* electrocardiogram, *MSE* multiscale entropy, *SE* standard error).

The presented findings established that in the young, the intrinsic SAN mechanisms mainly contribute to the long-range patterns and low frequencies within the beat intervals, while the ANS mainly contributes periodic regulation and short-term variance. Thus, in attempt to identify the intrinsic SAN signature (i.e. represented by the ABK state), the LF and HF bands were filtered (see “Methods” for details). The filter was trained on data from 5 patients and tested on data from 5 patient. MSE analysis of the effect of this method showed a good fit between the ABK and the filtered data (Fig. 6A; average MSE p-values were 0.35 and 0.31 among MSE scales for train and test groups, respectively).

The presented findings established that intrinsic SAN mechanisms in the adult contribute to both long- and short-range BIV. Moreover, the anesthesia contributes PSD in the HF band. In attempt to identify the intrinsic SAN signature, we filtered bands in LF and VLF bands that were not dominate by the intrinsic SAN and the HF to decipher the ANS effect. The filter was trained with data from 9 patients and tested with data from 5 patients. MSE analysis of the effect of this method showed a good fit between the ABK and the filtered data among MSE scales (Fig. 6B; average MSE p-values were 0.65 and 0.49 across all scales for train and test groups, respectively). Taken together, such filters can be used to assess intrinsic SAN function based on basal ECG data.

## Discussion

In the present study, existing BSL dataset of patients without known history of cardiac diseases including sinus dysfunction was used to investigate the age-dependent relative contribution of the ANS and intrinsic SAN mechanisms to SAN function in humans. In young patients, the intrinsic mechanisms and ANS mainly contributed to long- and short-term BIV, respectively. In contrast, in the adult, the ANS contributed to short-term BIV, while intrinsic mechanisms contributed to both short- and long-term BIV. Thus, supporting the first hypothesis, there is an age-dependent shift in the relative contribution of the ANS and the intrinsic SAN mechanisms to SAN function. Anesthesia affected ANS function in young patients and both intrinsic mechanisms and ANS in adults, supporting the second hypothesis that anesthesia affects the ANS and intrinsic SAN mechanisms in an age-dependent manner. Finally, this work demonstrated that features of the intrinsic mechanisms can be calculated from BIs obtained in-vivo, achieving similar results as drug intervention, supporting the 3^rd^ hypothesis specific features, which represent the “signatures” of the ANS and intrinsic SAN mechanisms in BI signals can be obtained from BIV analysis of standard electrocardiogram (ECG) recordings.

In both young and adult patients, the ANS contributed to short-term BIV. Similar evidence has been reported in dogs, rabbits and mice^18^. A similar conclusion was reached in atrial fibrillation patients with no contribution of intrinsic SAN mechanisms (SAN is silent)^11^. In contrast, an age-dependent shift was noted for contribution of the intrinsic SAN mechanisms. In young patients, the intrinsic mechanisms contributed mainly to long-term BIV, while in the adult, the intrinsic mechanisms contribute to both short- and long-term BIV. Thus, in the adult, changes in short-term BIV can be the result of deterioration of one or both of the systems. Age-dependent changes in the sympathovagal balance and increased intrinsic SAN beating variability were documented here as was documented before in mice^25^.

Because the test cannot be performed in young patients while awake, the ECG recordings was performed in the presence of propofol and dexmedetomidine also before ABK state. The double-blockade experiments in both young and adult patients were performed under anesthesia. The data showed that propofol and dexmedetomidine affected ANS function in young patients and both intrinsic mechanisms and ANS in the adult. Thus, Anesthesia affects BIV, particularly average heart rate, in an age-dependent manner.

In the current work, administration of both propofol and dexmedetomidine anesthesia led to prolongation of the BI, with no effect on total power. Additionally, under these conditions, the HF band increased and the VLF band decreased in young patients. Tarvainen et al. reported that both propofol and dexmedetomidine lead to a LF band decrease and HF band increase, while the effect of dexmedetomidine was higher^26^. In contrast, both Deutschman et al.^27^ and Galletly et al.^28^ found that propofol decreased total power, LF, and HF PSD content, with no change in BI. However, their experiments were performed with ventilation. Kanaya et al.^29^ measured a reduction in the HF band, with no change in BI. However, the bispectral index (BIS) was not maintained under their experiments compared to our conditions. Our conclusion that the SAN mechanisms are also affected by anesthesia, is supported by former findings^30^.

Under ABK together with anesthesia, we found a linear relationship between intrinsic heart rate (IHR) and age that followed the equation: IHR = 113–(0.54 * Age). Jose and Collison^7^ reached an equation of IHR = 118.1–(0.57 * Age) based on results of an autonomic blockade clinical trial in healthy volunteers (n = 432) without anesthesia. The similarity between the two equations underscores the validity of our results and suggests that the intrinsic heart rate test is insensetive at least to propofol and dexmedetomidine anesthesia. Our IHR results and equation aligned with other clinical trials performed on different experimental sets and populations (Strobel et al.^31^, Marcus et al.^32^, Alboni et al.^33^, Cumming and Mir^34^ and Opthof^35^). Moreover, as was shown in mice^36^, while the basal HR is not age-dependent, IHR declined with age.

We performed similar experiments in dog^11^, mice and rabbits^18^. Interestingly, the contribution of ANS and intrinsic SAN mechanisms to SAN function in young patients was similar to that measured in anesthetized rabbits. Because experiments are first performed on animals, it is important to choose the most appropriate animal model that best presents change in both average BI and in BIV, and similar underlying mechanisms. Animal experiments also shed light on the mechanisms that lead to an age-dependent shift in the contribution of ANS and intrinsic SAN mechanisms to BI dynamics^37^. Frailty, which was quantified in young and aged mice using a frailty index (FI), was greater in aged vs. young mice. Increases in FI were associated with prolonged SAN recovery time, reduced conduction velocity, increased interstitial fibrosis and alterations in expression of matrix metalloproteinases. Thus, both age-associated functional and morphological changes affect SAN function.

We suggest here a set of filters which can mimic anesthesia or BI of ABK. Their application can simulate the ABK conditions without need for ANS blockade. The approach is based on frequency analysis and was validated by MSE. Because the relative contribution of ANS and intrinsic SAN mechanisms to SAN function is aged-dependent, we generated an aged-dependent filter. Other filters can be chosen, as needed, when future analyses are performed. Note that the filter was applied on anesthetized data for the young and thus is a “pure” ANS blockade, while in the adult, it filtered both anesthesia and ANS blockade.

### Limitations

This retrospective analysis of existing data from daily clinical practice, may have introduced a bias in patient selection. Future experiments on volunteers without any background cardiac condition will overcome this limitation.

Temporary blockade of the ANS requires anesthesia in young patients because they can’t sit still. As was shown here, the anesthesia affects the ANS system and its effect was age-dependent. Future experiments on nonanesthetized adults are necessary to confirm our conclusion regarding the effect of anesthesia on BI dynamics.

Our designed ANS blockade filter assumed that ANS and intrinsic SAN mechanisms are the two main mechanisms contributing to SAN. Other factors can also contribute to BIV, e.g. hormone level in blood^32^. Future experiments with isolated SAN tissue or isolation of the hormone effect are expected to determine their relative contributions. Note that in dogs^9^, rabbits and mice^16^, these factors have minimal effects as compared to those of the ANS and SAN systems.

Here we blocked the cholinergic and beta-adrenergic receptors to mimic the effect of ANS blockade. However, atropine and propranolol only partly block ANS effects on SAN, as there are additional receptors (e.g. NPY receptors, galanin receptors, neurokinin receptors, VIP receptors) that mediate these effects^38,39^. Note however that the effect of ANS blockade with these drugs on HRV was similar to the effect of spinal cord injury^40^. Thus, one may claim that the majority of ANS effect on HRV is from cholinergic and beta-adrenergic receptors. Because the guideline allows only the use of atropine and propranolol, future clinical testing of other drugs, specifically in young subjects, will enhance our understanding of the relative contribution of other receptor antagonist on heart rate dynamics.

## Methods

### Data recording and pre-processing

Electrocardiography was performed on 39 patients with paroxysmal supraventricular tachycardia prior to ablation (see patient details in Table S3). The data acquisition and the study experimental protocol were carried out in accordance with relevant guidelines and regulations of the clinical electrophysiology laboratory at Saitama Medical University International Medical Center. All experimental protocols were approved by Saitama Medical University International Medical Center review board (#2021-070). Informed consent was obtained from all subjects and/or their legal guardians. Patients with unstable vital signs, inconsistent QRS morphology during a time epoch (such as intermittent accessory pathway) or frequent premature contractions (> 5%) were excluded. Standard 12-lead surface ECG was recorded at a sampling rate of 977 Hz and exported by CardioLab version 6.9.0 (GE healthcare Japan, Japan). Because of the morphology in which Q, QRS, and T waves can be easily separated, Lead II, V1 or V5 was chosen for analysis. In addition, in some patients, a standard 3-channel Holter ECG recorder (Fukuda Denshi, Japan) was used to record baseline ECG at a 128 Hz sampling rate.

In adults, 10 min baseline ECG was recorded at room temperature at the beginning of an ablation session (“baseline” or BSL). Patients were awake, in a supine position. This was followed by introduction and maintenance of deep sedation with a combination of propofol (a bolus injection of 2mg/kg followed by 4mg/kg/h continuous infusion) and dexmedetomidine (6 µg/kg/min during the first 10 min, then maintained at 0.6 µg/kg/min). A BIS monitor was used to adjust the sedation depth (target BIS 40–60). An oral airway device and capnometer were used to secure and monitor spontaneous breathing. When a stable > 10 min segment was available following the establishment of steady state in anesthesia, ECG was recorded for 10–15 min. At the end of ablation, an electrophysiology test was performed to assess intrinsic properties of the heart. As a part of this process, dual autonomic blockade was induced by simultaneous injection of propranolol (0.2 mg/kg) and atropine (0.04 mg/kg)^14^; vital signs were vigorously monitored throughout. With the exception of average BI, there were no noticeable changes in vital signs such as arterial blood pressure, BIS level, breathing or blood oxygenation. A 10–15 min stable segment was recorded at this stage. There were no adverse events associated with either ablation or autonomic blockade.

The procedure was identical in young patients, however, BSL recording was not performed. Due to the difficulty of taking a > 10 min segment of ECG in an awake state on a table, 24-h Holter ECG was performed at around the same time as the BSL recording. The 30 min segment at the beginning of the Holter ECG recording was excluded and the subsequent 30 min segment was used for analysis (cut into 3 windows of 10 min each). For recordings collected in young and adult patients under anesthesia, up to 3 min at the beginning of the record was cut out to avoid a transient segment. For ABK records in both young and adult patients, the 2 min segment at the beginning of the record was cut out to avoid a transient segment. If the mean BI was higher in ABK than BSL or if the HF peak of the PSD was not decreased or abolished in ABK relative to BSL, the data were excluded.

### RR interval time-series processing

The time period between two consecutive R-peaks, defined as RR interval time-series, was calculated. The R-peak detection and RR interval calculation were performed using the open-source software PhysioZoo^19^, which allows analysis of ECG recordings of human and other mammalian data. PhysioZoo was used for ECG peak detection and segmentation, calculation of RR intervals, ectopic beat removal and calculation of BIV metrics. Please refer to the supplemental material published by Behar et al.^19^, which contains comprehensive descriptions of the algorithms and their implementation details. The PhysioZoo platform is freely available at https://physiozoo.com.

### RR interval time-series construction and filtering

Three techniques were used to remove the non-physiological data from the RR intervals: (1) range-based filtering, which discards intervals longer or shorter than threshold values (RRmin = 0.28 s and RRmax = 2.4 s, which correspond to heart rates of 214 bpm and 50 bpm, respectively), (2) moving-average filtering, which removes intervals different from their neighboring intervals by 20% with a window size of 21 samples (10 samples on each side of the central sample) and (3) quotient filtering, which eliminates an interval if it varies from its previous or successive interval by 20%.

### BIV measures calculation

After processing, RR intervals were used to calculate BIV measures using the PhysioZoo platform^19^. These measures include linear, frequency and non-linear (entropy) analysis. Please refer to the supplemental material of the PhysioZoo paper^19^, which contains comprehensive descriptions of BIV matrices.

### Filter design

Anesthesia and ANS filters were applied on the processed RR interval time-series. We used finite impulse response (FIR) band-stop filters. Each filter range was adjusted according to the band power content and peak PSD of the frequency bands. Single or dual range filters were used and the filter order was defined as 100 and 150 for mono and dual band filters, respectively. “Blackman” tapering window^41^ of 101 and 151 was used for the single and dual band filters, respectively. For young patients, we used a dual FIR filter as the anesthesia filter, with a first range of 0.013–0.03 Hz applied on a segment of VLF band; and a second range of 0.046–0.158 Hz applied on all LF bands. For adult patients, a mono FIR filter was used as the anesthesia filter, with a range of 0.03–0.2 Hz applied on a segment of the VLF band, all LF bands, and a segment of the HF band. For young patients, a mono FIR filter was used as the ABK filter, with a range of 0.05–0.58 Hz applied on most of LF band and almost all HF bands. For adult patients, a dual FIR filter was used as the ABK filter, with a first range of 0.015–0.14 Hz applied on most of VLF and LF bands and a second range of 0.16–0.58 Hz applied on almost all HF bands.

### Statistics

Descriptive statistics are presented as mean ± SE. To analyze the repeated measures data (patients were measured in the basal state, under anesthesia, and under autonomic blockade) a one-way mixed ANOVA model was used with a random term for patient. The model tests for differences in the mean among the six groups (the two age groups, each in 3 states). Post hoc pairwise comparisons are computed to determine which groups differ with a false discovery rate (FDR) adjustment to the p-values. Significant differences among the pairs are presented in Fig. 3 and Tables S1 and S2.

SAS program was used for statistical tests and Matlab to generate the programs.

### Supplementary Information

Supplementary Tables.

## Data Availability

All codes implemented in the BIV analysis algorithms are freely available at https://physiozoo.com. A link to the datasets will be made available upon request from Assoc. Prof. Yael Yaniv (yaely@bm.technion.av.il) following publication of the paper.
