# Supplementary material for: Age-dependent contribution of intrinsic mechanisms to sinoatrial node function in humans
Source: Sci Rep. 2023 Nov 1;13:18875. doi: 10.1038/s41598-023-45101-7 (PMC10620402; doi:10.1038/s41598-023-45101-7)
Supplement: Supplementary file 1 — Supplementary Tables. [file 41598_2023_45101_MOESM1_ESM.docx]

**Tables**

**Table S1: Beat interval variability for basal (BSL), anesthesia, and autonomic blockade states in the presence of anesthesia (ABK) for young and adult patients.** Frequency domain measures were based on Welch modeling, and frequency band normalization was calculated as relative percentage of band summation. Results are reported as mean ± SE. (BI, beat interval, STD, standard deviation, RMSSD, root mean square of successive differences (SD) of BI, pNN50, the percentage of BI which differs by at least 50 ms from their preceding intervals, SEM, standard error of the mean BI, SD, BI interval standard deviation along the perpendicular to the line-of-identity or line-of-identity for SD1 and SD2, respectively, HF, high-frequency band, LF, low-frequency band, VLF, very-low-frequency-band, norm., normalized, MSE, multiscale entropy). ^*^compared to same age BSL, ^#^compared to same age Anesthesia. Significant comparisons are based on FDR adjusted p-values from post-hoc pairwise comparisons from the mixed ANOVA models.

|  | Units | Young | | | Adult | | |
| --- | --- | --- | --- | --- | --- | --- | --- |
|  |  | BSL (n=14) | Anes. (n=16) | ABK (n=10) | BSL (n=23) | Anes. (n=11) | ABK (n=17) |
| Mean BI | (ms) | 679.65±34.73 | 986.55±43.09 ^*^ | 572.37±18.38 ^*,#^ | 873.58±24.9 | 1115.19±38.94 ^*^ | 731.8±26.96 ^*,#^ |
| STD BI | (ms) | 53.74±7.37 | 54.19±2.93 | 6.18±0.9 ^*,#^ | 36.92±2.93 | 28.16±5.53 | 11.48±2.58 ^*,#^ |
| RMSSD | (ms) | 26.92±3.88 | 73.31±4.68 ^*^ | 2.56±0.64 ^*,#^ | 19.28±2.84 | 28.35±5.54 | 6.35±1.28 ^*,#^ |
| pNN50 | (%) | 8.84±3.08 | 53.71±4.18 ^*^ | 0±0 ^*,#^ | 4.06±2.36 | 11.06±4.19 | 0.33±0.29 ^#^ |
| SEM | (ms) | 1.84±0.26 | 2.24±0.14 | 0.19±0.03 ^*,#^ | 1.42±0.12 | 1.26±0.26 | 0.41±0.1 ^*,#^ |
| SD1 | (ms) | 19.05±2.74 | 51.88±3.31 ^*^ | 1.81±0.45 ^*,#^ | 13.64±2.01 | 20.07±3.92 | 4.49±0.91 ^*,#^ |
| SD2 | (ms) | 73.17±10.23 | 55.08±3.99 ^*^ | 8.45±1.26 ^*,#^ | 50.07±3.82 | 33.69±7.09 ^*^ | 15.45±3.55 ^*,#^ |
| Total Power (welch) | (ms^2^) | 2504.55±587.48 | 2405.85±304.89 | 11.18±2.16 ^*,#^ | 1021.89±201.57 | 791.23±403.9 | 69.37±36.34 ^*^ |
| HF Power (welch) | (ms^2^) | 340.84±82.86 | 1691.21±186.47 ^*^ | 3.28±1.41 ^#^ | 200.99±74.27 | 309.74±112.17 | 25.74±9.51 |
| LF Power (welch) | (ms^2^) | 611.55±126.98 | 348.58±88.74 ^*^ | 0.14±0.03 ^*,#^ | 257.28±56.2 | 143.66±84.43 | 11.75±9.02 ^*^ |
| VLF Power (welch) | (ms^2^) | 1552.15±429.9 | 366.06±116.51 ^*^ | 7.76±1.58 ^*^ | 563.62±96.11 | 337.83±239.6 | 31.88±21.45 |
| HF norm. | (n.u.) | 16.25±2.4 | 72.03±4.25 ^*^ | 26.15±8.45 ^#^ | 16.87±2.49 | 53.08±5.12 ^*^ | 56.71±5.77 ^*^ |
| LF norm | (n.u.) | 27.64±2.13 | 12.93±1.88 ^*^ | 1.19±0.19 ^*,#^ | 23.78±2.16 | 16.44±3.16 ^*^ | 6.11±1.98 ^*,#^ |
| VLF norm | (n.u.) | 56.1±2.99 | 15.05±3.36 ^*^ | 72.66±8.43 ^*,#^ | 59.35±2.7 | 30.49±5.13 ^*^ | 37.18±5.43 ^*^ |
| alpha1 | (n.u.) | 1.23±0.05 | 0.45±0.05 ^*^ | 0.59±0.06 ^*^ | 1.17±0.05 | 0.61±0.06 ^*^ | 0.44±0.06 ^*^ |
| alpha2 | (n.u.) | 0.97±0.04 | 0.65±0.05 ^*^ | 1.42±0.07 ^*,#^ | 1±0.03 | 0.81±0.1 ^*^ | 1.32±0.06 ^*,#^ |
| Beta (welch) | (n.u.) | -0.83±0.1 | -0.37±0.14 ^*^ | -2.55±0.13 ^*,#^ | -1.15±0.11 | -0.68±0.2 ^*^ | -1.67±0.16 ^*,#^ |
| MSE5 | (n.u.) | 1.49±0.07 | 1.12±0.06 ^*^ | 0.45±0.07 ^*,#^ | 1.34±0.07 | 1.06±0.11 ^*^ | 0.33±0.09 ^*,#^ |
| MSE10 | (n.u.) | 1.58±0.07 | 0.98±0.08 ^*^ | 0.48±0.07 ^*,#^ | 1.45±0.05 | 1.02±0.07 ^*^ | 0.3±0.1 ^*,#^ |
| MSE15 | (n.u.) | 1.55±0.07 | 0.94±0.09 ^*^ | 0.64±0.09 ^*^ | 1.52±0.08 | 1.07±0.12 ^*^ | 0.3±0.12 ^*,#^ |
| MSE20 | (n.u.) | 1.64±0.1 | 0.8±0.08 ^*^ | 0.72±0.11 ^*^ | 1.54±0.11 | 1.13±0.13 ^*^ | 0.3±0.1 ^*,#^ |

**Table S2: Beat interval variability of young versus adult patients in basal (BSL), anesthesia, and autonomic blockade in the presence of anesthesia (ABK) states.** Significant comparisons are based on FDR adjusted p-values from post-hoc pairwise comparisons from the mixed ANOVA models. (BI, beat interval, STD, standard deviation, RMSSD, root mean square of successive differences (SD) of BI, pNN50, the percentage of BI which differs by at least 50 ms from their preceding intervals, SEM, standard error of the mean BI, SD, BI interval standard deviation along the perpendicular to the line-of-identity or line-of-identity for SD1 and SD2, respectively, HF, high-frequency band, LF, low-frequency band, VLF, very-low-frequency-band, norm., normalized, MSE, multiscale entropy).

|  | Units | BSL Young vs.  BSL Adult | Anes. Young vs. Anes. Adult | ABK Young vs.  ABK Adult |
| --- | --- | --- | --- | --- |
| Mean BI | (ms) | <0.001 | 0.015 | 0.0037 |
| STD BI | (ms) | 0.0031 | <0.001 | - |
| RMSSD | (ms) | - | <0.001 | - |
| pNN50 | (%) | - | <0.001 | - |
| SEM | (ms) | - | <0.001 | - |
| SD1 | (ms) | - | <0.001 | - |
| SD2 | (ms) | 0.0037 | 0.023 | - |
| Total Power (welch) | (ms^2^) | 0.0015 | 0.0022 | - |
| HF Power (welch) | (ms^2^) | - | <0.001 | - |
| LF Power (welch) | (ms^2^) | 0.0022 | - | - |
| VLF Power (welch) | (ms^2^) | <0.001 | - | - |
| HF norm. | (n.u.) | - | 0.013 | <0.001 |
| LF norm | (n.u.) | - | - | - |
| VLF norm | (n.u.) | - | 0.033 | <0.001 |
| alpha1 | (n.u.) | - | - | - |
| alpha2 | (n.u.) | - | - | - |
| Beta (welch) | (n.u.) | - | - | <0.001 |
| MSE5 | (n.u.) | - | - | - |
| MSE10 | (n.u.) | - | - | - |
| MSE15 | (n.u.) | - | - | 0.037 |
| MSE20 | (n.u.) |  | - | 0.023 |

**Table S3: Patient information.** WPW, Wolf-Parkinson-White; AVNRT, atrioventricular nodal reentrant tachycardia; NS, statistically non-significant.

|  | All (n=39) | Young (n=16) | Adult (n=23) | P value (Young vs Adult) |
| --- | --- | --- | --- | --- |
| Age | 39±26 | 16±11 | 59±12 | <0.0001 |
| Female (%) | 64.1% (25) | 40% (7) | 73% (18) | <0.05 |
| Type of arrhythmia |  |  |  |  |
| WPW syndrome | 38.4% (15) | 68.8% (11) | 18.0% (4) | <0.005 |
| AVNRT | 48.7% (19) | 23.0% (3) | 69.6% (16) | <0.005 |
| Atrial flutter | 5.1% (2) | 13.3% (2) | 0 | NS |
| Others | 7.6% (3) | 0 | 13.0% (3) | NS |
| Comorbidities |  |  |  |  |
| Hypertension | 10.3% (4) | 0 | 17.3% (4) | NS |
| Dyslipidemia | 5.1% (2) | 0 | 8.6% (2) | NS |
| Diabetes | 0 | 0 | 0 | NS |
| Heart Failure | 0 | 0 | 0 | NS |
| Antiarrhythmic drug use on the test day |  |  |  |  |
| Beta blocker | 5.1% (2) | 0 | 8.6% (2) | NS |
| Other | 0 | 0 | 0 | NS |
